# Atrial septal defect in a patient with congenital disorder of glycosylation type 1a: a case report

**DOI:** 10.1186/s13256-017-1528-4

**Published:** 2018-01-24

**Authors:** Ruo-hao Wu, Dong-fang Li, Wen-ting Tang, Kun-yin Qiu, Yu Li, Xiong-yu Liao, Dan-xia Tang, Li-jun Qin, Bing-qing Deng, Xiang-yang Luo

**Affiliations:** 1 Department of Paediatrics, Sun Yat-sen Memorial Hospital, Sun Yat-sen University, Guangzhou, 510120 People’s Republic of China; 2 Key Laboratory of Malignant Tumor Gene Regulation and Target Therapy of Guangdong High Education Institutes, Sun Yat-sen University, Guangzhou, 510120 People’s Republic of China; 3 Department of Cardiology, Sun Yat-sen Memorial Hospital, Sun Yat-sen University, Guangzhou, 510120 People’s Republic of China; 4 Department of Research and Molecular Diagnostics, Cancer Center of Sun Yat-sen University, Sun Yat-sen University, Guangzhou, 510060 People’s Republic of China

**Keywords:** Congenital disorders of glycosylation type 1a, Congenital heart defects, Atrial septal defects, Spontaneous closure

## Abstract

**Background:**

Atrial septal defect often become more severe when encountered in genetic syndromes. Congenital disorder of glycosylation type 1a is an inherited metabolic disorder associated with mutations in *PMM2* gene and can affect almost all organs. Cardiac abnormalities vary greatly in congenital disorder of glycosylation type 1a and congenital heart defects have already been reported, but there is little knowledge about the effect of this inherited disorder on an existing congenital heart defect. Herein we report for the first time on a baby with congenital disorder of glycosylation type 1a with atrial septal defect and make a comparison of changes in atrial septal defect by follow-ups to the age of 3.

**Case presentation:**

Our patient was an 8-month-old Han Chinese boy. At the initial visit, he presented with recurrent lower respiratory infection, heart murmur, psychomotor retardation, inverted nipples, and cerebellar atrophy. Echocardiography revealed a 8 mm secundum atrial septal defect with left-to-right shunt (Qp/Qs ratio 1.6). Enzyme testing of phosphomannomutase 2 demonstrated decreased levels of phosphomannomutase 2 activities in fibroblasts. Whole exon sequencing showed he was heterozygous for a frameshift mutation (p.I153X) and a missense mutation (p.I132T) in *PMM2* gene. The diagnosis of congenital disorder of glycosylation type 1a with atrial septal defect was issued. Now, he is 3-years old at the time of this writing, with the development of congenital disorder of glycosylation type 1a (cerebellar atrophy become more severe and the symptom of nystagmus emerged), the size of atrial septal defect increased to 10 mm and the Qp/Qs ratio increased to 1.9, which suggested exacerbation of the atrial septal defect. Congenital heart defect-associated gene sequencing is then performed and shows there are no pathogenic mutations, which suggested intrinsic cardiac factors are not the cause of exacerbation of the atrial septal defect in our patient and it is reasonable to assume congenital disorder of glycosylation type 1a can worsen the situation of the existing atrial septal defect.

**Conclusions:**

This report highlights the view that congenital disorders of glycosylation type 1a should be excluded when faced with congenital heart defect with cerebellar atrophy or neurodevelopmental delay, especially when the situation of congenital heart defect becomes more and more severe.

## Background

Atrial septal defect (ASD) is anatomically characterized by a defective interatrial septum, which allows communication between the left and right sides of the heart. ASD represents 30 to 40% of congenital heart defects (CHDs) and is the third most common type of CHD. Most ASD is sporadic with no specific cause and often encountered in genetic syndromes such as Noonan syndrome, Holt–Oram syndrome, and Down’s syndrome. The natural history of isolated atrial communications varies depending on anatomical type, defect size, and patient-specific factors. According to previously studies, spontaneous closure occurs frequently in young patients (diagnosis at younger than 1 year) with small defects (size ≤ 8 mm) [1]. However, in many patients with a genetic syndrome with ASD, defects do not close spontaneously, most of them remain unchanged, even increased, especially in those mutations in genes essential to cardiac septation such as Holt–Oram syndrome (*NKX2-5* mutation) [2].

Congenital disorders of glycosylation (CDG), which were first recognized in the 1980s, are a group of inherited metabolic disorders caused by defects in the synthesis of glycans and processing of their attachment to proteins and lipids. More than 20 types of CDG have been reported. Congenital disorder of glycosylation type 1a (CDG-1a, OMIM #212065) is the most common type in this group, also known as phosphomannomutase 2 (PMM2) deficiency (OMIM #601785), which is associated with a mutation in *PMM2* gene, resulting in the defective synthesis of N-linked oligosaccharides, sugars linked together in a specific pattern and attached to proteins or lipids [3].

Patients with CDG-1a can be divided into four stages by ages: infantile multisystemic disability, childhood ataxia-intellectual disability, teenage leg atrophy disability, and adulthood stable disability. In the first stage of CDG-1a, two distinct clinical forms are important to note: non-fatal neurologic form and neurologic-multivisceral [4]. The first one is characterized by strabismus, psychomotor retardation, hypotonia, and cerebellar hypoplasia, besides that, inverted nipples, abnormal fat distribution, and feeding problems are common in this form. The second one is a multiorgan disorder which can cause a great number of abnormalities, almost all organs can be involved; hepatopathy, diarrhea, nephritic syndrome, pericardial effusion, cardiomyopathy, coagulopathy, and multiorgan failure are often observed in this form and its abnormalities are still broadening [5].

Among those abnormalities, cardiac involvements vary greatly in CDG-1a. CHD [6], pericardial or cardiac effusions [7], cardiomyopathies (both hypertrophic and dilated cardiomyopathies) [8], and transient myocardial ischemia [9] have been reported in CDG-1a. CHDs have been rarely reported in CDG-1a and all reported cases are cardiac arteriovenous defects [6]. According to previously studies, the cause of CHD in CDG-1a may be attributed to abnormal neural crest migration and differentiation caused by lack of glycosylated proteins in the embryonic period and this view has been well confirmed in *in vitro* and animal experiments [10]. However, to the best of our knowledge, knowledge about the effect of CDG-1a on an existing CHD is scarce.

Here we report for the first time on a baby with CDG-1a with ASD, who has no family history of CHD or mutations in genes essential to cardiac septation such as *NKX2-5*, *GATA4*, and *TBX5*. This patient is followed up to 3-years old and a comparison of the changes in defect size and blood shunt of ASD has been made.

## Case presentation

Our patient was an 8-month-old Han Chinese baby boy who was referred for heart murmur, recurrent lower respiratory infections, psychomotor retardation, and hypotonia at his initial visit. He was the first child delivered to healthy, non-consanguineous young parents of Han Chinese origin, and was born at 39 weeks’ gestation with a birth weight of 3.1 kg after an uneventful pregnancy. He had no specific family medical history. During the neonatal period, hypotonia and feeding difficulties were noted and recurrent lower respiratory tract infections at 3 months of age (once per month). When he was 8-months old, he presented with heart murmur, failure to thrive, psychomotor retardation (unable to raise his head by himself), and hypotonia. A physical examination showed low weight (only 4 kg) with growth retardation, hypotonia with diminished deep tendon reflexes, bilateral alternating squint, inverted nipples, and hepatomegaly (the lower edge of his liver was located 3 cm below the costal margin at the mid-clavicular line), besides that, we noted a soft (grade 2/6) systolic ejection murmur at the second and the third left intercostal space with a diastolic rumble over his left lower sternum and fixed splitting of S2 was also noted. The rest of the physical examination was unremarkable.

**Fig. 1:**
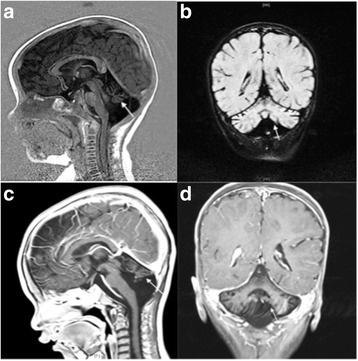
**a** At our patient’s first visit (8-months old), cranial magnetic resonance imaging showed severe atrophy of cerebellar hemispheres; coronal position (*arrow*). **b** Severe hypoplasia of cerebellar vermis was noted at sagittal position on cranial magnetic resonance imaging (*arrow*). **c** When he was 3-years old, the atrophy of cerebellar hemispheres still existed (*arrow*) and the volume of cerebellar hemispheres was evidently less than the average level of his peers. **d** The absence of cerebellar vermis was noted obviously (*arrow*)

**Fig. 2:**
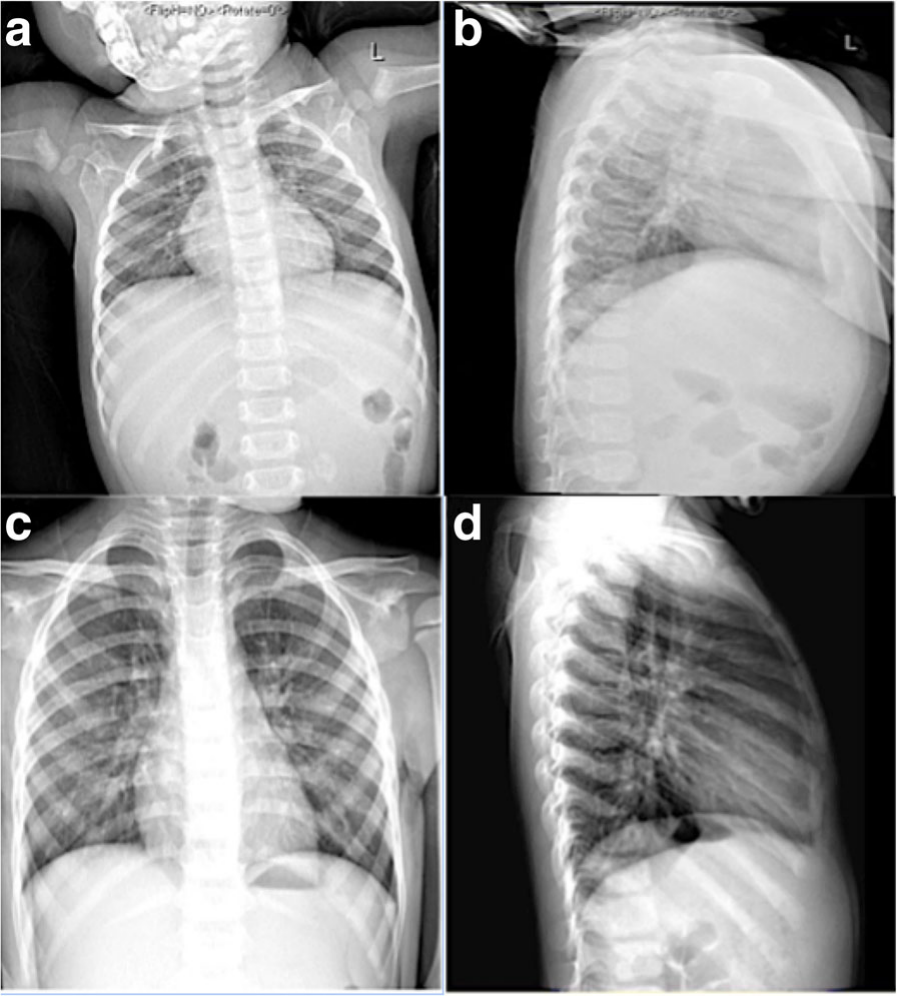
**a** At our patient’s first visit (8-months old), chest radiography showed narrowing of aortic knob and enlargement of right atrial at the frontal position. **b** At the same time, pulmonary blood stasis and increase of lung markings were also found at the lateral position, those image features may have been caused by atrial septal defect. **c** When he was 3-years old, narrowing of aortic knob and enlargement of right atrial were improved slightly compared with his first visit. **d** However, at the lateral position, increase of lung markings and pulmonary blood stasis were obviously aggravated compared with his first visit, those image features were attributed to the deterioration of atrial septal defect

**Fig. 3:**
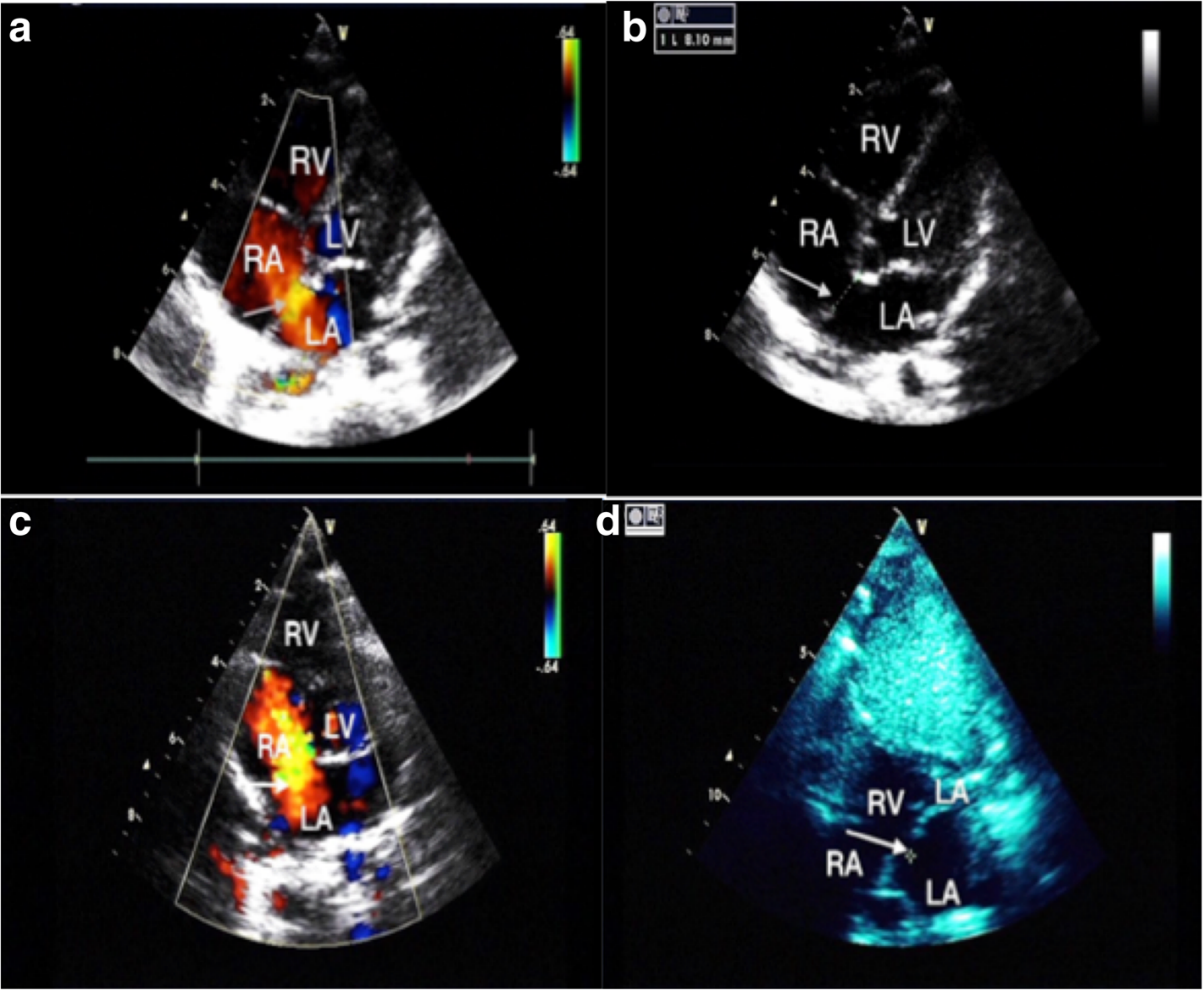
**a** At our patient’s first visit (8-months old), color Doppler echocardiography showed a secundum defect with left-to-right shunt in the middle of atrial septum and the Qp/Qs ratio was 1.6 which meant the blood shunt was moderate (*arrow*). **b** On transthoracic echocardiography, we noted that the defect size of atrial septal defect was 8.1 mm (*arrow*). **c** When he was 3-years old, the left-to-right shunt still existed and the Qp/Qs ratio was 1.9 which meant the blood shunt was moderate to severe (*arrow*). **d** On transthoracic echocardiography, we noted that the existing defect had increased and the size was 10 mm (*arrow*), the blood shunt and defect size were all increased, which suggested exacerbation of atrial septal defect. *LA* left atrium, *LV* left ventricle, *RA* right atrium, *RV* right ventricle

Routine laboratory investigations showed normal urine analysis and fecal analysis, normal complete blood count and blood glucose, and normal blood gas analysis. Immunological examinations showed hypoimmunoglobulinemia (immunoglobulin G (IgG), 3 g/L; control values, 7 to 16 g/L) and low percentage of CD4^+^ T lymphocytes (CD4^+^T%, 15%; control values, 32 to 51%) in our patient. Biochemical tests revealed his alanine aminotransferase (ALT) was significantly elevated at 450 IU/L (control values, 9 to 50 IU/L) and aspartate aminotransferase (AST) at 329 IU/L (control values, 15 to 40 IU/L), alpha fetal protein (AFP) was markedly elevated at 115.8 ng/ml (control values, ≤ 25 ng/ml), and creatine kinase (CK) levels were mildly elevated at 240 IU/L (control values, 26 to 174 IU/L). An endocrine metabolic workup revealed low levels of insulin-like growth factor 1 in serum (<25 ng/ml; control values, > 55 ng/ml), the rest of endocrine metabolic parameters including insulin, thyroxine, 25-hydroxy vitamin D3 and ammonia, acylcarnitine profile, and lactate were normal. Coagulation parameters showed activated partial thrombin time was prolonged by more than 1.5 times (57.7 seconds; control values, 23 to 35 seconds); antithrombin III was significantly decreased at 34.1% (control values, 85 to 130%). Cranial magnetic resonance imaging (MRI) demonstrated severe atrophy of cerebellar hemispheres with vermis hypoplasia (Fig. 1a, b). Chest radiography showed pulmonary blood stasis and increase of lung markings at the frontal position, narrowing of the aortic knob, and enlargement of right atrial at the lateral position; those image features were considered to be caused by ASD (Fig. 2a, b). Transthoracic echocardiography revealed normal thickness of interventricular and dilation of the right ventricle. Besides these, a secundum ASD in the middle of the atrial septum (defect size, 8.1 mm) was noted. Color Doppler echocardiography showed a secundum defect with left-to-right shunt in the middle of the atrial septum and the Qp/Qs ratio was 1.6 which meant the blood shunt was moderate (Fig. 3a, b). Transabdominal ultrasound showed hepatomegaly (left subcostal oblique diameter, 73.6 mm) with heterogenous echoes, and we considered that it was caused by fatty liver. Urinary ultrasonography showed no abnormal results in bilateral kidneys, ureters, and bladder.

**Fig. 4:**
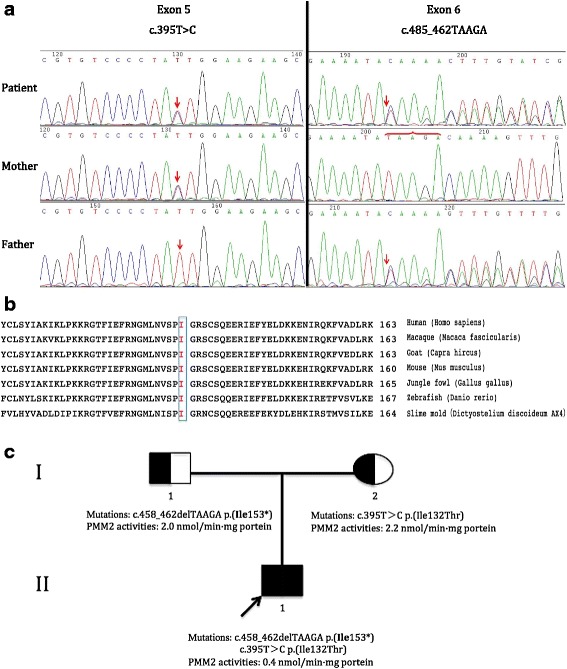
**a** Direct gene sequencing analysis of *PMM2* gene revealed the substitution of T for C at position 395 in exon 5, this mutation existed in the patient and his mother (*right*); another mutation was the absence of TAAGA at position 458_462 in exon 6, this mutation presented in the patient and his father (*left*). **b** Cross-species comparison of phosphomannomutase 2 protein sequence showed that the isoleucine (I) residue at amino acid position 132 was conserved from protists to primates (shown by the *box*), which suggested that mutation in this amino acid position may affect the normal structure and function of phosphomannomutase 2. **c** With pedigree analysis, we found that the patient’s father was heterozygous for the I153X mutation with normal phosphomannomutase 2 activities and his mother was heterozygous for the I132T mutation with normal phosphomannomutase 2 activities, while our patient (*arrow*) was a proband in his family and carried two mutations derived from his parents with decreased levels of phosphomannomutase 2 activities. *PMM2* phosphomannomutase 2

With the above clinical results, CDG-1 was considered, and isoelectric focusing of serum transferrin analysis was performed, this analysis showed markedly increased levels of disialotransferrin band and decreased levels of tetrasialotransferrin band, this transferrin pattern was consistent with CDG-1. Enzyme testing of cultured skin fibroblasts for PMM2 activities in our patient demonstrated decreased levels of PMM2 activities (0.4 nmol/min · mg protein; control values, 1.8 to 8.8 nmol/min · mg protein), while the PMM2 activities in his father and mother were both normal (2 and 2.2 nmol/min · mg protein, respectively). The diagnosis of CDG-1a was obvious. Chromosomal microarray analysis was performed and found no chromosomal rearrangement. Whole exon sequencing was performed after his parents gave their informed consent for this genetic analysis and the result showed that our patient was heterozygous for the frameshift mutation and missense mutation in *PMM2* gene (Fig. 4a). The first was 458_462 del TAAGA mutation in exon 6, which was predicted to result in the premature translational termination at amino acid position 153 (I153X), causing the absence of the 93 amino acids of C-terminal domain, and further led to no activity of PMM2 protein derived from this allele. The second was 395 T>C mutation in exon 5, which was predicted to result in the amino acid exchange p.I132T. The p.I132T mutation has been reported to be pathogenic [11]. Furthermore, a cross-species comparison of the PMM2 protein sequence revealed that this isoleucine residue at amino acid position 132 was conserved from protists to primates (Fig. 4b) and, thus, was likely to be functionally important. Analysis of parental blood samples showed that our patient’s father was heterozygous for the I153X mutation and his mother was heterozygous for the I132T mutation (Fig. 4c).

After admission, our patient was treated regularly with general supportive treatment including: liver, heart, and muscle protection; anticoagulant therapy and anti-infective treatment; and intravenously administered immunoglobulin infusion in order to improve immunity. Now, at the age of 3-years old failure to thrive, bilateral alternating squint and inverted nipples still exist, and his weight only increased to 10 kg; the location and feature of existing cardiac souffle remain unchanged, but fixed splitting of S2 disappeared. His hepatomegaly is resolved (the lower edge of his liver was located 1 cm below the costal margin at the mid-clavicular line) and he can raise his head by himself for minutes and speak several single words (two to three words), such as ma-ma or pa-pa. Hypotonia gradually improved but he is still unable to sit or crawl. The infections of his lower respiratory tract became more severe than before and the frequency increased to two to three times per month. Besides that, the symptom of nystagmus emerged which is thought to be related to the aggravation of cerebellar atrophy. Hypoimmunoglobulinemia and low percentage of CD4^+^ T lymphocytes remained (IgG, 4.7 g/L; CD4^+^T%, 20%). Liver dysfunction and abnormalities of CK and CK–myocardial band (CK-MB) improved (AST 80 IU/L and AFP 37 ng/ml, CK 100 IU/L and CK-MB 20 IU/L); the levels of antithrombin III slightly increased (45%). A re-examination MRI of his cerebellar revealed atrophy of cerebellar hemispheres still exists and the volume of cerebellar hemispheres was evidently less than the average level of his peers; the absence of cerebellar vermis was noted obviously (Fig. 1c, d). These results may suggest the state of CDG-1a in our patient progresses continually and may develop to the next stage (childhood ataxia-intellectual disability). Re-examination of chest radiography showed narrowing of the aortic knob and enlargement of right atrial had improved slightly compared with his first visit, but pulmonary blood stasis was obviously more aggravated than before (Fig. 2c, d). Re-examination of echocardiography revealed the existing defect had increased and the size increased from 8 to 10 mm; the left-to-right shunt also existed and the Qp/Qs ratio increased from 1.6 to 1.9, which means the blood shunt was more severe than before (Fig. 3c, d). The blood shunt and defect size were all increased, which suggested the exacerbation of ASD. Then, we performed CHD-associated gene sequencing in our patient and his family, which contained mutations in 24 common genes essential to cardiac septation (*ACTC1, BRAF, CRELD1, ELN, G6PC3, GATA4, GDF1, GJA1, HRAS, JAG1, KCNJ2, KRAS, MYH6, NKX2-5, NRAS, PTPN11, RAF1, RBM10, SOS1, TBX1, TBX20, TBX5, TLL1, ZFPM2*). Mutations in those genes may cause exacerbation of ASD; however, we found that our patient and his parents have no pathogenic mutations in those genes.

## Discussion

Research already proves that cardiomyopathies, both hypertrophic and dilated cardiomyopathies, have been associated with almost all types of CDG-1, especially in CDG-1a, and are the most prevalent cardiac abnormality in CDG-1a [8]. As previously reported [12], CDG-1a can increase the severity of cardiomyopathies and accelerate their progress by disturbing epicardial–myocardial cell interactions, finally causing many patients with CDG-1a to die of heart failure. By contrast, CHD is a rare complication in CDG-1a and knowledge about the development and progression of CHD in CDG-1a is scarce.

In our patient, we found the situation of ASD had been deteriorating with the development of CDG-1a through a comparison of before and after treatment and observation. It seemed that CDG-1a can worsen the situation of ASD, but we still could not exclude the possibility that the exacerbation of ASD was caused by intrinsic cardiac factors directly; then, we performed CHD-associated gene sequencing in our patient and his parents. The result of CHD-associated gene sequencing demonstrated that our patient and his parents have no mutations in genes encoding formation and development of cardiac septation and it is reasonable to assume CDG-1a can worsen the situation of existing ASD to some extent, but the mechanism is still unknown.

The spontaneous closure of ASD is a very complicated process and the mechanism is still unclear, it is generally believed that low weight gain with growth retardation, persistent continuous blood shunting, and delayed development of the cardiac septum may affect this process, causing the exacerbation of ASD [13]. Immune dysfunction and failure to thrive are both common in patients with CDG-1a [14]. In our patient, immune dysfunction and failure to thrive occurred at an early stage and persisted in the whole course of the disease. Immune dysfunction can aggravate infections of the lower respiratory tract, causing the exacerbation of pulmonary blood stasis, and finally promote the persistence of blood shunting. Failure to thrive can cause low weight gain with growth retardation, which not only retards the process of ASD size reduction by affecting the growth of the thoracic cavity, but also delays the development of the cardiac septum by restricting the intake of protein. Furthermore, CDG-1a, as one of N-glycosylation disorders, can cause the disturbance of epicardial–myocardial cell interactions, which may also influence the development of the cardiac septum [12]. These factors may be responsible for the exacerbation of ASD in our patient, but more studies would be required in order to confirm it.

## Conclusions

In conclusion, we present the first case of CDG-1a with ASD and assume CDG-1a can worsen the situation of existing CHD. We feel from this report and along with a previous report that CDG-1a cannot only cause CHD, but also aggravate the situation of existing CHD. It should be excluded from the diagnosis of CDG-1a when faced with CHD with cerebellar atrophy or neurodevelopmental delay, especially when the situation of CHD becomes more and more severe.

## Abbreviations

AFP: Alpha fetal protein
ALT: Alanine aminotransferase
ASD: Atrial septal defect
AST: Aspartate aminotransferase
CDG: Congenital disorders of glycosylation
CDG-1a: Congenital disorder of glycosylation type 1a
CHD: Congenital heart defect
CK: Creatine kinase
CK-MB: Creatine kinase–myocardial band
MRI: Magnetic resonance imaging
PMM2: Phosphomannomutase 2

## References

[1] Geva T, Martins JD, Wald RM. Atrial septal defects. Lancet. 2014;383(9932):1921–32. 10.1016/S0140-6736(13)62145-5 24725467

[2] Chin J, Pereira S, Camacho A, Pessoa B, Bento D, Amado J, *et al.* Holt-Oram syndrome: a case report. Rev Port Cardiol. 2014;33(11):737. 10.1016/j.repc.2014.06.005 25455949

[3] Jaeken J. Congenital disorders of glycosylation (CDG): it’s (nearly) all in it! J Inherit Metab Dis. 2011;34(4):853–8. 10.1007/s10545-011-9299-3 21384229

[4] Stefanits H, Konstantopoulou V, Kuess M, Milenkovic I, Matula C. Initial diagnosis of the congenital disorder of glycosylation PMM2-CDG (CDG1a) in a 4-year-old girl after neurosurgical intervention for cerebral hemorrhage. J Neurosurg Pediatr. 2014;14(5):546–9. 10.3171/2014.7.PEDS14102 25192236

[5] Funke S, Gardeitchik T, Kouwenberg D, Mohamed M, Wortmann SB, Korsch E, *et al.* Perinatal and early infantile symptoms in congenital disorders of glycosylation. Am J Med Genet A. 2013;161A(3):578–84. 10.1002/ajmg.a.35702 23401092

[6] Romano S, Bajolle F, Valayannopoulos V, Lyonnet S, Colomb V, de Barace C, *et al.* Conotruncal heart defects in three patients with congenital disorder of glycosylation type Ia (CDG Ia). J Med Genet. 2009;46(4):287–8. 10.1136/jmg.2008.057620 19357119

[7] Kristiansson B, Stibler H, Conradi N, Eriksson BO, Ryd W. The heart and pericardial effusions in CDGS-I (carbohydrate-deficient glycoprotein syndrome type I). J Inherit Metab Dis. 1998;21(2):112–24. 10.1023/a:1005387408009 9584262

[8] Footitt EJ, Karimova A, Burch A, Yayeh T, Dupre T, Vuillaumier-Barrot S, *et al.* Cardiomyopathy in the congenital disorders of glycosylation (CDG): a case of late presentation and literature review. J Inherit Metab Dis. 2009;32 Suppl 1:S313–9. 10.1007/s10545-009-1262-1 19757145

[9] Marquardt T, Hulskamp G, Gehrmann J, Debus V, Harms E, Kehl HG. Severe transient myocardial ischaemia caused by hypertrophic cardiomyopathy in a patient with congenital disorder of glycosylation type Ia. Eur J Pediatr. 2002;161(10):524–7. 10.1007/s00431-002-1029-2 12297897

[10] Maschhoff KL, Baldwin HS. Molecular determinants of neural crest migration. Am J Med Genet. 2000;97(4):280–8. 10.1002/1096-8628(200024)97:4<280::aid-ajmg1278>3.0.co;2-n 11376439

[11] Grünewald S, Schollen E, Van Schaftingen E, Jaeken J, Matthijs G. High Residual Activity of PMM2 in Patients’ Fibroblasts: Possible Pitfall in the Diagnosis of CDG-Ia (Phosphomannomutase Deficiency). Am J Hum Genet. 2001;68:347–54. 10.1086/318199 PMC1235268 11156536

[12] Luo Y, Frances AH, Jonathan AE, Glenn LR. N-cadherin is required for neural crest remodeling of the cardiac outflow tract. Dev Biol. 2006;200(2):517–28. 10.1016/j.ydbio.2006.09.003 PMC1866362 17014840

[13] Lin KM, Liang CD, Chien SJ, Lin YJ, Lin IC, Lo MH, *et al.* Predictors for regression of large secundum atrial septal defects diagnosed in infancy. Acta Cardiol Sin. 2013;29(1):82–7. PMC4804964 27122688

[14] Garcia-Lopez R, de la Morena-Barrio ME, Alsina L, Perez-Duenas B, Jaeken J, Serrano M, *et al.* Natural killer cell receptors and cytotoxic activity in phosphomannomutase 2 deficiency (PMM2-CDG). PLoS One. 2016;11(7), e0158863. 10.1371/journal.pone.0158863 PMC4944953 27415628

